# Response of Wheat Storage Proteins and Breadmaking Quality to Dimethylpyrazole-Based Nitrification Inhibitors under Different Nitrogen Fertilization Splitting Strategies

**DOI:** 10.3390/plants10040703

**Published:** 2021-04-06

**Authors:** Ximena Huérfano, José-María Estavillo, Miren K. Duñabeitia, María-Begoña González-Moro, Carmen González-Murua, Teresa Fuertes-Mendizábal

**Affiliations:** Department of Plant Biology and Ecology, University of the Basque Country UPV/EHU, Apdo. 644, E 48080 Bilbao, Spain; jm.estavillo@ehu.eus (J.-M.E.); miren.dunabeitia@ehu.eus (M.K.D.); mariabegona.gonzalez@ehu.eus (M.-B.G.-M.); carmen.gmurua@ehu.eus (C.G.-M.); teresa.fuertes@ehu.eus (T.F.-M.)

**Keywords:** DMPP, DMPSA, dough extensibility, dough strength, gliadins, glutenins

## Abstract

Improving fertilizer nitrogen (N) use efficiency is essential to increase crop productivity and avoid environmental damage. This study was conducted during four crop cycles of winter wheat under humid Mediterranean conditions (Araba, northern Spain). The effects of N-fertilization splitting and the application of the nitrification inhibitors (NIs) 3,4-dimethylpyrazole phosphate (DMPP) and 2-(3,4-dimethyl-1H-pyrazol-1-yl) succinic acid isomeric mixture (DMPSA) as strategies to improve grain quality were examined. The hypothesis of this study was to test if the partial ammonium nutrition and the reduction of fertilizer losses presumably induced by the application of NIs can modify the grain gliadin and glutenin protein contents and the breadmaking quality (dough rheological properties). Among both NIs assayed, only DMPP showed a slight effect of decreasing the omega gliadin fraction, following splitting either two or three times, although this effect was dependent on the year and was not reflected in terms of dough extensibility. The slight decreases observed in grain quality in terms of dough strength and glutenin content induced by DMPP suggest that DMPSA is more promising in terms of maintaining grain quality. Nonetheless, these poor effects exerted by NI application on grain quality parameters did not lead to changes in the quality parameters defining the flour aptitudes for breadmaking.

## 1. Introduction

Wheat is one of the most important global crops. According to FAO [1], this crop provides around 757 million tons. Close to 65% of the harvested wheat is destined for human consumption in many forms, of which bread is one of the most important end uses. Given its predominance in human diets, cultivated wheat has to meet the specific quality criteria for the manufacture of the wide range of food products derived from it [2]. The most important determinant of wheat breadmaking quality is the amount of grain protein and its composition, which are determined by both genetics and environment factors [3]. Grain proteins can be broadly divided into structural/metabolic and storage proteins [4]. The latter are important quality determinants because they are responsible for dough quality. In mature grain, the storage proteins, gliadins, and glutenins, are aggregated in polymers with different sizes and solubility, and their distribution and solubility play a critical role in governing wheat flour properties [5,6]. Gliadins include three types of proteins with distinct sequences, referred to as α + β, γ, and ω gliadins. Glutenins consist of low and high molecular weight (LMW and HMW) subunits. HMW-glutenins subunits have the largest effect on breadmaking quality [7]. LMW-glutenins have less impact on dough quality, and the roles of these proteins are less clear [8]. Gliadins and glutenins form a network during dough processing, giving the unique property of viscoelasticity to the dough. The relative composition of these proteins has important effects on gluten behavior and therefore on the functional properties of the dough [9]. It is generally accepted that gliadins confer dough extensibility (L) and glutenins are responsible for dough strength (W) [10].

The environmental conditions and the use of different agronomic practices can have a greater influence on wheat grain nitrogen (N) content, and consequently on grain protein content, than genetic or varietal differences. Among agronomic practices, N-fertilization is considered by many as the main factor affecting storage proteins and therefore, influencing wheat breadmaking quality [11]. N-fertilization is conventionally applied at two stages: at tillering and stem elongation [12]. However, Gate [13] and Oury et al. [14] proposed the application of a third application at the “flag leaf just visible” stage, to enrich the N content of the vegetative organs, so more N would be available to be translocated later to the grain, leading to an increase in the final grain protein content. Hence, splitting fertilizer application may also greatly affect the composition of the grain protein fraction [15,16]. A third N application has become a common farm practice in many regions in northern Europe. However, under Mediterranean climatic conditions, this has led to very different results regarding the grain N content [17,18,19,20,21].

Wheat is usually cultivated under intensive agriculture systems, requiring high inputs of N fertilizers, which produce negative environmental impacts such as nitrate (NO_3_^−^) leaching or N gaseous loses by nitrification, denitrification and, ammonia volatilization [22,23]. To avoid or minimize these N losses, new molecules as nitrification inhibitors (NIs) have been developed to delay the oxidation of ammonium (NH_4_^+^). At present, 3,4-dimethylpyrazole phosphate (DMPP) is one of the NIs widely used in agriculture [24,25]. Recently, the 2-(3,4-dimethyl-1H-pyrazol-1-yl) succinic acid isomeric mixture (DMPSA) (CA 2933591 A12015/06/18 Patent) was synthesized to allow its combination with other fertilizers, such as calcium ammonium nitrate (CAN) or diammonium phosphate (DAP), due to its non-polarity. Many studies have demonstrated that the use of NIs significantly decreases the risk of NO_3_^−^ leaching in a wide range of soil types [26,27,28,29,30,31,32] and N_2_O gaseous emissions [33,34,35,36,37]; thus, the application of NIs should allow crop plants to absorb more available N. In fact, in order to mitigate the climate change by lowering N losses from agriculture, the Intergovernmental Panel on Climate Change (IPCC) has proposed the use of ammonium-based fertilizers accompanied by nitrification inhibitors as a possible tool. Nevertheless, their application leads to partial ammonium nutrition in plants that can influence both crop yield and quality. Different results have been observed in relation to this; many studies have reported no changes in wheat yield using DMPP [26,38,39], whereas others reported slight increases in wheat yield [40,41]. Thus, the utility of NIs has been demonstrated for an environmental purpose, maintaining or slightly increasing the yield. Nevertheless, partial NH_4_^+^ nutrition induced by the application of NIs can also have physiological effects that could affect grain quality. Related to this, Fuertes-Mendizábal et al. [42] showed an improvement in grain quality under greenhouse conditions when NH_4_^+^ was the exclusive N source. Very little research has been done in relation to the effect of NI application on wheat grain quality. A study by Guardia et al. [43] reported slightly different results in grain quality after DMPSA application in a single fertilization application; and other studies [26,31,40] have described no changes in grain N content when different NIs are applied. Therefore, the challenge in this study was to evaluate the effect of the use of DMPP and DMPSA in combination with the effect of splitting the N fertilizer application on wheat quality, analyzing the composition of the grain storage proteins and the dough rheological parameters.

## 2. Results

### 2.1. Wheat Yield and Grain N Content

**Figure 1:**
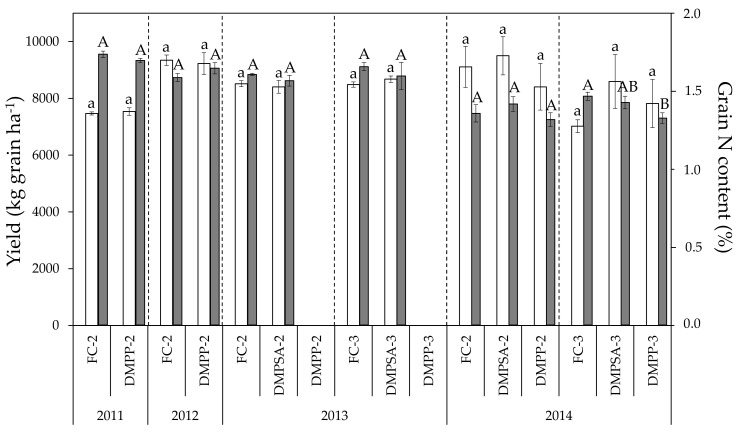
Yield (light bars) and grain N content (dark bars) in each crop cycle. For each parameter, treatments sharing the same letter (lowercase and uppercase letters for yield and grain N content, respectively) within each crop cycle and splitting treatment (separated by discontinue lines) do not differ significantly at *p* < 0.05.

Wheat yield ranged between 7020 and 9499 kg ha^−1^ during the four crop cycles studied (Figure 1). As shown in Table 1, the factor year significantly affected grain yield, whereas the splitting of fertilizer exerted a slight effect, which was reflected in the significant decrease of about 23% in grain yield in FC-3 with respect to FC-2 treatment only in 2014 (Figure 1). NI application did not influence grain yield. The effect size of the year on grain N concentration was much higher, with grain N concentration values ranging from 1.32% to 1.74% (Figure 1). The splitting effect on grain N content was evaluated in 2013 and 2014, when the Student’s *t*-test comparison between the N-fertilization in two and three amendments within each N-fertilizer treatment (AS (ammonium sulphate), DMPP and DMPSA) revealed no significant differences. Therefore, the splitting of a rate of 180 kg N ha^−1^ had no effect on grain N content. Only a slight decrease in N concentration was observed in 2014 when DMPP was split three times (DMPP-3 vs. FC-3); this decrease was not observed with the application of DMPSA (Figure 1).

### 2.2. Breadmaking Quality

**Figure 2:**
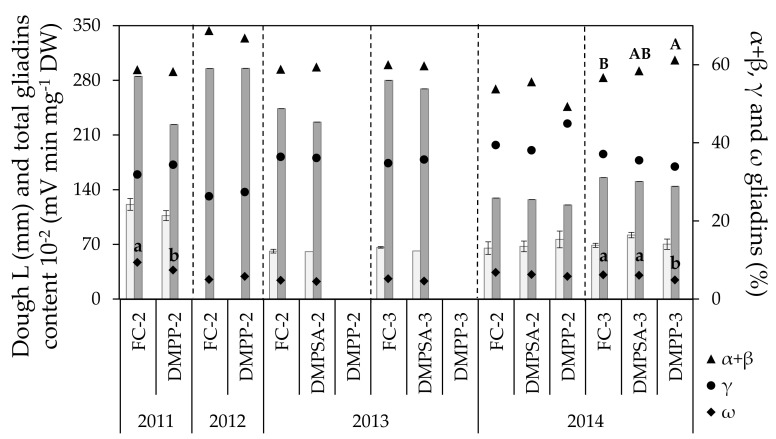
Dough extensibility (L, light bars), total grain gliadin content (represented as the total gliadin content divided by 100, dark bars) and grain gliadin fractions (α + β gliadins, γ gliadins and ω gliadins) in each crop cycle. Treatments sharing the same letter (uppercase and lowercase letters for α + β and ω gliadins, respectively) within each crop cycle and splitting treatment (separated by discontinue lines) do not differ significantly at *p* < 0.05.

**Figure 3:**
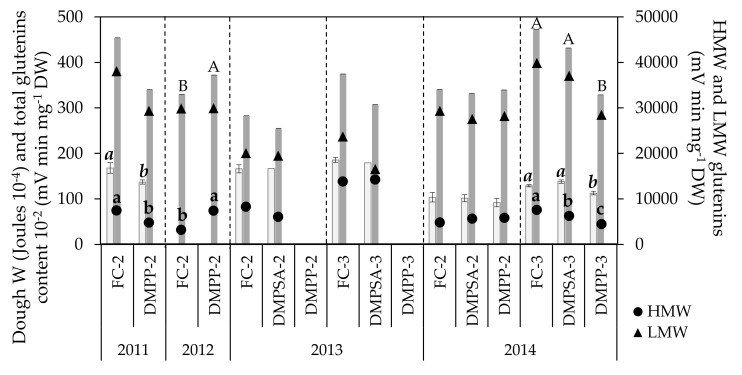
Dough strength (W, light bars), total grain glutenin content (represented as the total glutenin content divided by 100, dark bars) and grain fractions of high molecular weight (HMW) and low molecular weight (LMW) glutenins in each crop cycle. Treatments sharing the same letter (lowercase, cursive, and uppercase letters for HMW-glutenins, dough strength, and total glutenin content, respectively) within each crop cycle and splitting treatment (separated by discontinued lines) do not differ significantly at *p* < 0.05.

During the whole study period, values for dough L ranged between 60.75 and 120.75 mm (Figure 2). No effect of N-fertilization splitting or NI application was observed on this parameter (Table 1, Figure 1). As was the case for grain N content, extensibility values obtained in 2011 were higher than those of 2013 and 2014, but no changes were observed in L as a result of NI application, regardless of the year, the splitting of the N dose, or the inhibitor applied. Values observed for dough W ranged from 91.2 × 10^−4^ to 185 × 10^−4^ joules (Figure 3), a significant decrease of around 36% being observed for all N treatments in 2014 with respect to 2013 (Figure 2). The effect of splitting was evaluated in 2013 and 2014, and only in 2014 was an increase observed, of around 28% in W values in the plots for which N was split three times compared to plots for which N was split two times. Regarding the effect of NIs, a significant W decrease of 18% occurred when applying DMPP split two times (DMPP-2 vs. FC-2) in 2011, and a significant decrease of 13% when applying DMPP split three times (DMPP-3 vs. FC-3), in 2014. It must be emphasized that DMPSA application did not have any effect on the dough W.

### 2.3. Grain Storage Proteins

In general, most of the effect on gliadin content was due to the changing conditions from one year to other (Table 1). The total gliadin content did not change as a consequence of N-fertilization splitting or NI application, but only varied between years according to the differences in grain N content (Figure 1 and Figure 2). The effect of splitting and NI application was not consistent across all of the crop cycles evaluated. The γ gliadin fraction remained stable regardless of the splitting or NI applied. By comparison, NI application showed a slight but significant effect on the ω gliadin fraction; a significant decrease in this fraction was observed with DMPP application in 2014 under N-fertilization split three times (DMPP-3 vs. FC-3), with a concomitant increase in the α + β gliadin fraction. In addition, a decrease in the ω gliadin fraction was also observed in 2011 with DMPP application under N-fertilization split twice (DMPP-2 vs. FC-2).

The grain glutenin contents showed a small and year-dependent response to NI application (Figure 3). The proportion of variance explained by the splitting was greater for HMW-glutenins than for LMW-glutenins (Table 1). When DMPP was split twice, the HMW-glutenins content decreased significantly in 2011 (Figure 3), coinciding with the lower W described previously, whereas differences in the total glutenins content were non-significant. Nevertheless, in 2012 the opposite was observed, with DMPP-2 treatment showing a significantly higher HMW-glutenin content than FC-2, which was reflected in a significantly higher content of total glutenins. In contrast, the application of NIs split twice did not show any effect in 2013 and 2014 (Figure 3). When N-fertilization was split three times (in 2014), DMPP-3 significantly decreased the HMW-glutenin fraction by 41%, concomitantly with a significant decrease of around 27% in the total glutenin content and a similar decreasing trend in dough W. By comparison, DMPSA-3 induced a smaller decrease in the HMW-glutenin fraction of only 17%, which was not accompanied by a significant decrease in the total glutenin content or in dough W (Figure 3). Total glutenin and LMW-glutenin contents showed a lower response to splitting compared to HMW-glutenins (Table 1), as indicated by the considerably larger size effect of HMW-glutenins (ɳ^2^_p_ = 0.543). Similarly, this response was not consistent across all of the crop cycles evaluated. When NI applications were split three times compared to FC-2, data showed a similar trend to that observed for grain N content and W, showing a higher HMW-glutenin content in DMPSA-3 compared to FC-2, and a lower glutenin content in DMPP-3 with respect to the other treatments there were split three times.

The gliadin/glutenin ratio was maintained unchanged regardless of the N-fertilization treatment (Table 1).

### 2.4. Soil Mineral Nitrogen Content

**Figure 4:**
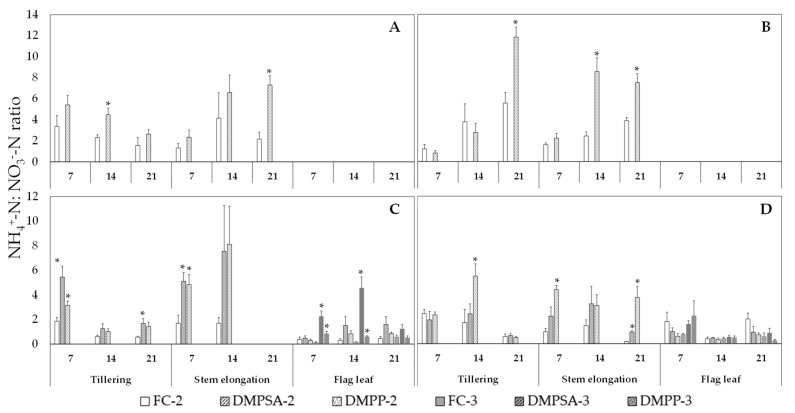
Ammonium: nitrate (NH_4_^+^-N:NO_3_^−^-N) ratio in 2011 (**A**), 2012 (**B**), 2013 (**C**) and 2014 (**D**). Values are presented 7, 14, and 21 days after each fertilization event (tillering, steam elongation, and flag leaf). An asterisk (*) indicates significant differences (*p* < 0.05) between DMPSA or DMPP and their respective fertilized control (FC-2 or FC-3) in each day and splitting treatment.

To evaluate the effect of NI application in delaying the oxidation of NH_4_^+^ to NO_3_^−^, the NH_4_^+^-N:NO_3_^−^-N ratio was determined weekly three times after each fertilization event (Figure 4). This ratio was higher in 72% of the evaluated moments in the treatments in which NIs were applied. Within this 72%, 43% of the cases were significantly higher. N fertilization splitting did not affect the NH_4_^+^-N:NO_3_^−^-N ratio, and the effect of DMPP and DMPSA on this ratio was similar.

## 3. Discussion

### 3.1. Nitrification Inhibitors and Breadmaking Quality

Wheat yield and grain N content were more affected by the year (environmental conditions) than by any of the N-fertilizer treatments (Table 1). In this sense, Guzman-Bustamante et al. [44] also recently reported that DMPP application has no effect on wheat yield and quality. Grain N content is the main quality parameter indicative of wheat breadmaking quality. In our study, a strong effect of the year was observed in grain N content, with significant differences between years observed. In 2012 the N content was significantly lower than that in 2011, and differences were more evident in 2014, when N contents were between 10% and 27% lower than those in 2013 (Figure 1). These lower grain N contents can be ascribed to an “N dilution” effect due to the high yields obtained in 2012 and 2014. Some authors have studied the potential improvement of grain quality by the application of NIs due to the increase in ammonium nutrition induced and to the reduction of the fertilizer-losses by leaching or by N_2_O emission [45], e.g., Peltonen and Virtanen [46] described an increase in grain N content using granular ammonium nitrate fertilizer stabilized with dicyandiamide (DCD). However, the results obtained in our study revealed that the use of both NIs split twice (DMPP-2 and DMPSA-2 treatments) had no effect on grain N content, which is in agreement with the results obtained by Villar and Guillaumes [40], Liu et al. [31], and Duncan et al. [47].

Although it has been described that an exclusively ammonium nutrition can lead to an increase in wheat grain quality [42], in our case, by applying NIs and favoring ammonium nutrition, no effect was observed in field conditions. Although DMPP and DMPSA have been proved to be effective in reducing gaseous N emissions to the atmosphere under humid Mediterranean conditions, the effect of these NIs on grain quality as a consequence of increasing the NH_4_^+^-N:NO_3_^−^-N ratio was found to be slight. Therefore, the higher/longer presence of NH_4_^+^ in NI treatments was not strong enough to be reflected in a higher grain N content at the end of the crop lifecycle. The climatic conditions of the study allowed an effective incorporation of the late N fertilizer application in GS37 to the soil–plant system, because the treatments that were split three times maintained a yield similar to that in the treatments split twice. Nevertheless, in the literature very different results have been reported regarding the effect of fertilization splitting on grain N content. In the same edaphoclimatic conditions of this study, under humid Mediterranean conditions, an improvement in grain protein content has been reported by a further splitting of the same N dose [19]. However, in the present study we did not observe a significant response of grain N content to the application of a third application (Figure 1), and the grain N content of FC-3 was found to be statistically equal to that of FC-2 in both crop cycles evaluated.

Although grain N content measurement discriminates between low and high protein flours, this protein content will not necessarily ensure that a given flour will have a satisfactory breadmaking quality [48]. Depending on both genetic and environmental factors, different flours with the same N content can have different rheological behavior and, therefore, different breadmaking quality. Although N-fertilizer rates and timing of application are decisive factors in the obtaining of not only high yields and increased protein content, but also improved alveogram parameters [49,50], there is a lack of results concerning the effect of NIs on these parameters. In our study, both L and W were found to be in consonance with the variations observed in grain N content among the different years. Thus, in general, dough L values obtained in 2011 were higher than those of 2013 and 2014, which agrees with the higher grain N content achieved that year. In fact, taking into account the entire experimental period, both variables were well correlated (r = 0.614, *p* < 0.01). Differences in dough W values between different years were also related with grain N content (r = 0.736, *p* < 0.01). In contrast, our results show that the N-fertilization treatments only induced an effect on W and not on L (Table 1), and only the year factor but not the N-treatments showed an effect on L. The W parameter varied significantly according to slight and non-significant changes in grain N content in 2011 and 2014 in response to fertilization management (Figure 2 and Figure 3). Regarding the effect of NIs, the fact that DMPSA application maintained unaltered W values, whereas the application of DMPP induced W reductions of 13% and 18% in DMPP-3 and DMPP-2 treatments, respectively, indicates that DMPSA might be a more promising NI in terms of breadmaking quality. However, we must emphasize that the decrease in W values registered after DMPP application did not change the flour classification in terms of breadmaking quality.

### 3.2. Nitrification Inhibitors and Grain Storage Proteins

Gliadins and glutenins conform the storage protein fraction of wheat grain and have been widely studied because they confer viscoelastic properties to wheat dough. The gliadin fraction consists of a heterogeneous mixture of simple polypeptide chains presenting a significant polymorphism. The gliadin fraction is involved in the quality determination due to this polymorphism, and to its amount and changes in the ratio between the different subgroups [5]. By comparison, glutenins are polymeric proteins whose effect on quality is determined by their abundance and composition, in addition to their polymerization degree [51]. It is well known that the type and the quantity of both protein fractions is determined genetically. However, the protein composition is also influenced by other factors such as N availability, temperature, or drought [52]. Fuertes-Mendizábal et al. [42] described changes in grain protein composition in ammonium-fed wheat plants. Therefore, we can expect changes in the protein quantity and/or its composition by NI application because these compounds not only maintain soil N as ammonium for a longer period of time, but can also reduce N losses by leaching and N_2_O emissions, thus increasing plants’ use efficiency of N. In the present study, grain total gliadin content showed the same trend as that observed for grain N content, and both parameters were well correlated (r = 0.740, *p* < 0.01). Thus, the low range of variation in grain N content led to a low range of variation in gliadin accumulation. In general, treatments with lower L values corresponded with lower gliadin content (Figure 2), and both variables were also well correlated (r = 0.414, *p* < 0.01). Hence, the absence of the response in terms of dough extensibility to the application of NIs, split either twice or three times, was supported by no changes in grain gliadin content (Figure 2). Moreover, the slight changes observed in the total gliadin content were attributable to the changes in the content of each gliadin fraction (Figure 2), because α + β, γ and ω gliadin fractions followed the same pattern as that of total gliadins. Altenbach et al. [53] proposed ω gliadins as the major source of protein variation in relation to environmental variability. This group of proteins belong to the sulfur-poor gliadins, whereas α, β and γ gliadins belong to the sulfur-rich gliadins [54]. A higher quantity of cysteine residues due to a higher proportion of α, β and γ gliadin fractions allows the formation of more disulfide bonds during the kneading of the dough. Thus, related to this, changes in the relative proportions between the different gliadins could lead to changes in the gluten characteristics, and therefore in grain quality. In fact, some studies have reported changes within the gliadin fraction in response to an increased N rate, but different results have been obtained among them. Daniel and Triboi [5] reported an increase in both ω and γ gliadins with a higher fertilizer rate, whereas α and β gliadins decreased. Wieser and Seilmeier [55] observed that ω gliadins increased more with an increase in N rate than α and γ gliadins. In our study, a decrease in the concentration of the ω gliadin fraction in fertilization without NIs and fertilization with DMPP application split twice or three times was observed, but this result was not consistent in all crop cycles evaluated. This decrease was compensated for by the α, β, and γ gliadin fractions, whose increases were not always found to be significant due to the fact that these fractions account for more than 90% of total gliadins. Thus, changes of the magnitude observed in the ω gliadins fraction were not enough to have an impact in terms of L and, therefore, these changes will have a low impact on the breadmaking quality. In this sense, Fuertes-Mendizábal et al. [42] described a decrease in the contribution of the ω gliadin fraction to the total gliadin content when plants were fed with ammonium as an exclusive N source. Therefore, in our case, it seems probable that the decrease in ω gliadin fraction described previously could be due to a higher presence of ammonium in the soil mediated by the NI application.

Although both gliadins and glutenins are responsible for dough viscoelastic properties, glutenins are related to both the elasticity and the extensibility, and, therefore, with the dough W [56]. HMW-glutenins are minor components of gluten in quantitative terms, but they are key factors in the process of dough development because they largely determine its elasticity [4]. In fact, the relative contribution of HMW-glutenins has been stated as an indicator of dough W [51]. The importance of these protein fractions is based on their influence on the nature of large glutenin polymers that confer elasticity and strength to dough made from wheat flour [53]. The total quantity of glutenin macropolymers in wheat grain is more sensitive to growth conditions than the grain protein content [57]. In our experiment, HMW-glutenins were well correlated with changes in the dough W (r = 0.669, *p* < 0.01), and they were affected not only by the year but also by the NI application (Figure 3). Variations in the plant nutritional status during the grain filling period can alter both the kinetics and the protein aggregate formation [58]. In this sense, a third late application would provide a better N status in advanced stages, favoring polymer formation and distribution, and potentially conferring better quality. However, this was not true for all years and both NIs. The slight changes observed in glutenin content had a poor effect on flour quality because, although significant changes in W were observed, the flour type in terms of quality did not change. This meant that the flour’s aptitude for breadmaking was the same for all of the treatments within the same year. The application of NIs did not further increase the total glutenin content, the HMW-glutenins content, or the dough W. In fact, a contrary effect was observed with the application of DMPP, which was not as marked as that of the application of DMPSA. Thus, as previously discussed, these results indicate that DMPSA might be a more promising NI in terms of breadmaking quality. However, results obtained in this experiment comparing the effect of DMPSA and DMPP on wheat quality are preliminary. To ascertain if this differential result among both NIs on breadmaking quality is consistent, more studies under a wider range of edaphoclimatic conditions and wheat varieties should be conducted.

## 4. Materials and Methods

### 4.1. Study Area and Experimental Design

**Figure 5:**
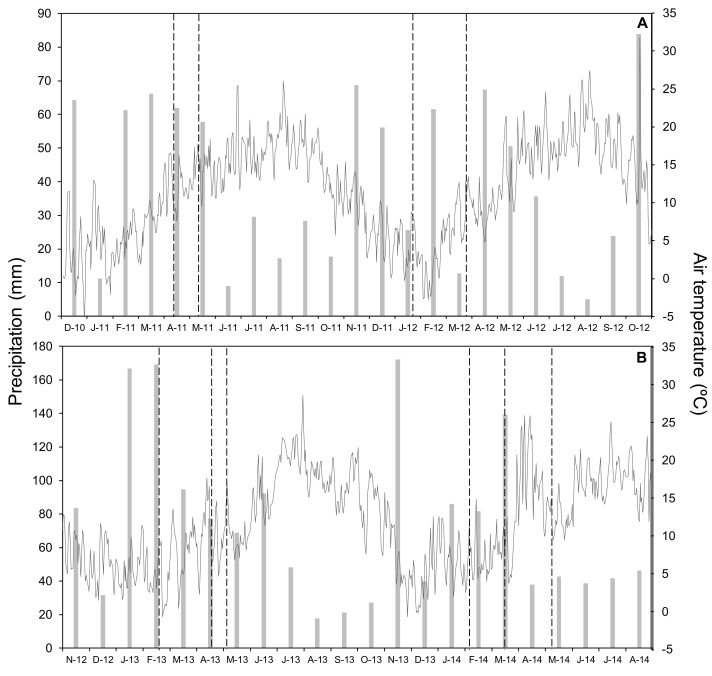
Monthly precipitation (bars) and daily mean air temperature (line) for the whole period of study in 2011 and 2012 (**A**) and in 2013 and 2014 (**B**). Vertical lines indicate N-fertilizer application.

This work consisted of four wheat crop cycles carried out in two nearby and representative localities of cereal production under the humid Mediterranean conditions of Araba (northern Spain). The first experimental field was Gauna (42°49′ N, 2°30′ W, and 580 m above sea level) and the second was Arkaute (42°51′ N, 2°37′ W, 530 m above sea level). In both locations the soil texture was clay loam (Table 2). In Gauna, the plots were supplied with 18 kg P ha^−1^ and 54 kg K ha^−1^ before seeding in 2010 and with 28 kg P ha^−1^ and 49 kg K ha^−1^ before seeding in 2011. The crop evaluated was winter wheat (*Triticum aestivum* L. Var. Cezanne), which was sown at a density of 220 kg seeds ha^−1^ on 16 December 2010 and 21 November 2011 in Gauna, and on 16 November 2012 and 11 November 2013 in Arkaute. Climatic conditions during all of the experimental period are presented in Figure 5.

All trials were designed as a randomized complete block experiment with four replicates and the area of each individual plot was 40 m^2^ (8 × 5 m). The total amount of N-fertilizer applied each year was 180 kg N ha^−1^, corresponding to the optimal level to obtain maximum grain yield in this region [59]. In 2011 and 2012, N-fertilizer was split into two applications at the stages of tillering (GS21) and stem elongation (GS30) according to the Zadoks scale (Table 3). In 2013 and 2014, N-fertilizer was split in two and three amendments at the grow stages GS21, GS30 and flag leaf (GS37). In 2011 and 2012, N-fertilizers used were ammonium sulphate nitrate (ASN 26%) (18.5% ammoniacal and 8.5 nitric) and the combination of ASN with DMPP, available in the market as ENTEC^®^ 26 (registered trademark of EuroChem Agro GmbH, Germany). In 2013 and 2014, N-fertilizers used were ammonium sulphate (AS 21%), the combination of AS with DMPP, available in the market as ENTEC^®^ 21 (registered trademark of EuroChem Agro GmbH, Germany), and the combination of AS with DMPSA. The rate of NIs (DMPP and DMPSA) in the N-fertilizer was 0.8% of the N in ammonium form. All of the treatment application dates and rates are detailed in Table 3. DMPP treatments of 2013 were excluded from the study due to a fertilization mistake in these plots.

### 4.2. Grain Yield and Quality Parameters

At the end of the crop lifecycle, a central surface of 12 m^2^ (1.5 × 8 m) per plot was harvested. The grain was separated from the straw to determine grain yield, which was expressed as adjusted to 12% moisture content. With regard to quality, grain was ground through a 1 mm screen (Micro Mill MM Rescht, Germany) for the determination of total N content by the Kjeldhal procedure (AOAC 1980) with a Kjeltec Auto sampler System 1035 analyzer (Tecator).

Quality dough parameters of strength (W), extensibility (L), and tenacity (P) were obtained through Chopin’s alveograph procedure (AACC) [60], except for the samples of year 2012, when the samples were degraded due to stocking technical problems. The determinations of dough P are not presented because this parameter was not affected by any of the N-fertilization treatments studied or the years evaluated.

### 4.3. Grain Storage Proteins Determination by Capillary Electrophoresis

The sequential extraction of each class of protein was the same as that of the Osborne method with modifications [61,62], according to Soba et al. [63]. Albumins and globulins were first removed with aqueous buffer. Gliadin extract was obtained with 1 mL 50% 1-propanol, vortexing for 30 min, and centrifuging at 15,000 rpm for 5 min. The remaining pellets were washed twice with 50% 1-propanol and supernatants were discarded. Glutenins were extracted by mixing the pellets with 1 mL of 50% 1-propanol containing 1% of dithiothreitol. After 1 h at 60 °C and centrifuging at 15,000 rpm for 10 min, the supernatant contained total glutenins. Gliadin and glutenin extracts were filtered with syringe filters (0.22 µm, Teknokroma) before analysis. HMW-glutenins were precipitated with acetone as described in Bean and Lookhart [61]. The supernatant contained LMW, and HMW were re-dissolved in 100 μL of 0.1% trifluoroacetic acid and 25% acetonitrile at 60 °C for 1 h. Protein extract stability was checked to allow their storage prior to analysis up to 24 h under refrigeration (4 °C) without obtaining any significant difference in their protein content. All reagents were analytical grade and solutions were prepared with ultrapure water obtained from a Milli Q water purification system (Millipore, Bedford, MA, USA).

Samples were analyzed by capillary electrophoresis using a Beckman^®^ 2100 P/ACE system controlled by System Gold Software version 810. The separation was performed using a silica uncoated capillary column (Polymicro Technologies, Phoenix, AZ, USA) with 27 cm length (20 cm to the detector) and 0.50 μm internal diameter. An isoelectric buffer containing 50 mM iminodiacetic acid, 0.05% hydroxy propyl methyl cellulose, and 20% acetonitrile was used [62,64]. Gliadins were separated at 45 °C and 15 kV, and glutenins were separated at 40 °C and 10 kV. Proteins were detected by UV absorbance at 214 nm with a photo diode array detector. Two replicates of each sample were prepared and each was measured in duplicate. As described Ronda et al. [64], to reduce the lack of reproducibility usually obtained in electrophoretic analysis, lysine-tyrosine-lysine tripeptide (Sigma Aldrich, Inc., St. Louis, MO, USA) was used as an internal standard. The area of each peak was calculated as the average of two replicates (injected in duplicate).

### 4.4. Soil Mineral Nitrogen Content

Soil nitrate and ammonium contents were determined by extracting 100 g fresh soil with 200 mL 1M KCl. Fresh soil was obtained from three soil sub-samples (2.5 cm diameter × 30 cm depth) taken in each plot 7, 14, and 21 days after each fertilizer application. The extracts were filtered through Whatman no. 1 filter paper to remove particles and, secondly through Sep-Pak Classic C18 Cartridges 125 Å pore size to eliminate organic carbon. The filtered solutions were used to determine the content of nitrate as described by Cawse (1967) [65] and the content of ammonium by the Berthelot method (Patton and Crouch, 1977) [66]. Finally, the NH_4_^+^-N:NO_3_^−^-N ratio was calculated.

### 4.5. Data Management

Data sets were analyzed for normality of standardized residuals using the Kolmogorov Smirnoff test and homogeneity of variances using Levene’s test. Analysis of variance (one-way ANOVA) and Student’s *t*-test were performed. Duncan’s multiple-range test was used to evaluate significantly different means. The effect size of each evaluated factor and their interactions was determined with the use of partial eta squared (ɳ^2^_p_), which describes a proportion of variability in a sample associated with an independent variable (Equation (1)):

ƞ^2^_p_ = SSeffect/(SSeffect + SSerror), (1)

where SSeffect is the sum of squares for the effect of interest and SSerror is the error term associated with this effect [67].

Relationships between different variables were tested by Spearman’s correlation.

All analyses were performed at a significance level *p* < 0.05 using IBM SPSS 21.0 statistical package (IBM Corp., Armonk, NY, USA).

## 5. Conclusions

This study demonstrates that NI application can only slightly alter wheat grain quality and protein composition, and these changes did not follow the same trend between years. Although more available N and a higher influence of ammonium on plant nutrition was expected by applying NIs, the changes observed were not substantial enough to reveal highly significant effects on flour quality that were consistent with the experiment. Changing edaphoclimatic conditions between years exerted a more significant effect. Two or three applications of NIs to the wheat crop maintained the grain yield, and did not induce strong changes in flour quality, in addition to the inherent changes ascribed to the N fertilizer splitting. Nor did they induce further changes in quality. Grain total gliadin content did not change due to NI application and, although a slight decrease in the relative fraction of ω gliadins was observed with DMPP, it was not reflected in terms of dough extensibility. Due to the slight decreases observed in grain quality in terms of dough strength and glutenin content induced by DMPP, DMPSA was found to be a more promising NI in terms of maintaining breadmaking quality. This, combined with the fact that dough strength was more sensitive than extensibility to NIs and glutenins were the protein fraction that showed the highest response to NI application, highlights the need to carry out further research focused on the effect of DMPP and DMPSA on the glutenin fraction.

## Figures and Tables

**Table 1:** Significance and size effect determined as partial eta-squared (ɳ^2^_p_) of each factor (year, splitting, and nitrification inhibitors (NIs)) and their interactions for the different variables measured. * *p* < 0.01, ** *p* < 0.05, *** *p* < 0.01. Gliadins (Gli) and glutenins (Glu).

	sig	ɳ^2^_p_	sig	ɳ^2^_p_	sig	ɳ^2^_p_	sig	ɳ^2^_p_	sig	ɳ^2^_p_	sig	ɳ^2^_p_
	Yield		Grain N		L		W		Gli		α and β-gli	
Year	***	0.230	***	0.789	***	0.611	***	0.670	***	0.735	***	0.767
Splitting	**	0.076	**	0.107	n.s.	0.028	**	0.221	**	0.087	**	0.111
NIs	n.s.	0.025	n.s.	0.018	n.s.	0.004	n.s.	0.032	n.s.	0.037	n.s.	0.300
Year × splitting	*	0.123	n.s.	0.000	n.s.	0.000	n.s.	0.002	n.s.	0.010	n.s.	0.000
Year × NIs	n.s.	0.007	n.s.	0.043	n.s.	0.090	n.s.	0.053	n.s.	0.063	n.s.	0.047
Splitting × NIs	n.s.	0.041	n.s.	0.023	n.s.	0.000	n.s.	0.003	n.s.	0.001	n.s.	0.000
Year × splitting × NIs	n.s.	0.011	n.s.	0.023	n.s.	0.000	n.s.	0.000	n.s.	0.000	n.s.	0.002
	γ-gli		ω-gli		Glu		LMW		HMW		Gli/Glu ratio	
Year	***	0.692	***	0.562	***	0.236	***	0.370	***	0.676	***	0.686
Splitting	n.s.	0.048	n.s.	0.004	***	0.168	*	0.066	*	0.543	n.s.	0.022
NIs	n.s.	0.026	*	0.057	*	0.070	*	0.064	n.s.	0.012	n.s.	0.010
Year × splitting	n.s.	0.010	n.s.	0.000	n.s.	0.001	n.s.	0.031	*	0.407	n.s.	0.009
Year × NIs	n.s.	0.044	*	0.157	n.s.	0.057	n.s.	0.030	n.s.	0.266	n.s.	0.032
Splitting × NIs	n.s.	0.004	n.s.	0.011	n.s.	0.020	n.s.	0.014	n.s.	0.025	n.s.	0.000
Year × splitting × NIs	n.s.	0.004	n.s.	0.009	n.s.	0.006	n.s.	0.000	*	0.141	n.s.	0.005

**Table 2:** Physical and chemical properties of the clay loam soil (0–30 cm depth) for each location.

	Soil Texture			Soil Chemical Properties								
Location	Sand (%)	Silt (%)	Clay (%)	pH	OM * (%)	N (%)	C:N	Carbonate (%)	P (ppm)	Ca (ppm)	Mg (ppm)	K (ppm)
**Gauna**	44	25	31	8.5	1.94	0.15	13.16	2.01	51.8	5695	87.5	128
**Arkaute**	36	28	36	8.4	2.9	0.23	12.60	2.01	106.0	12957	171.4	516

* OM, organic matter.

**Table 3:** Rates of fertilizer application (kg N ha^−1^) during each crop cycle at the different splitting dates that correspond to wheat growth stages of tillering, stem elongation, and flag leaf.

Treatment	Fertilizer Type	Tillering	Stem Elongation	Flag Leaf	Tillering	Stem Elongation	Flag Leaf
		**2011**			**2012**		
		14 March	11 April		24 January	26 March	
**FC-2**	ASN	60	120	0	60	120	0
**DMPP-2**	ASN + DMPP	60	120	0	60	120	0
		**2013**			**2014**		
		20 February	18 April	6 May	4 February	18 March	6 May
**FC-2**	AS	60	120	0	60	120	0
**FC-3**	AS	60	80	40	60	80	40
**DMPSA-2**	AS + DMPSA	60	120	0	60	120	0
**DMPSA-3**	AS + DMPSA	60	80	40	60	80	40
**DMPP-2**	AS + DMPP				60	120	0
**DMPP-3**	AS + DMPP				60	80	40

FC = fertilized control; ASN = ammonium sulphate nitrate; AS = ammonium sulphate; -2 = split twice; -3 = split three times.

## Data Availability

Our experimental data are not publishet neither available in any public source.
